# Systemic inflammatory indices for predicting severe placenta accreta spectrum: a retrospective cohort study

**DOI:** 10.7189/jogh.16.04353

**Published:** 2026-10-19

**Authors:** Xiafen Lu, Xiaoyun Lei, Congcong Guo, Qian Tang, Xiaomei Zhang, Wenting Mai, Ming Mo, Meiling Yuan, Wen Wang, Dazhi Fan

**Affiliations:** 1 Department of Obstetrics, The Affiliated Foshan Women and Children Hospital, Guangdong Medical University, Foshan, China; 2 Reproductive Medicine Center, The Affiliated Foshan Women and Children Hospital, Guangdong Medical University, Foshan, China; 3 Foshan Fetal Medicine Research Institute, The Affiliated Foshan Women and Children Hospital, Guangdong Medical University, Foshan, China; 4 Foshan Key Laboratory of Stem Cell and Maternal-Child Health, Foshan Women and Children Hospital, Foshan, China; 5 Stem Cell Clinical Research Center, Foshan Women and Children Hospital, Foshan, China; 6 Guangdong Medical University Stem Cell & Clinical Research Collaboration Alliance, The Affiliated Foshan Women and Children Hospital, Guangdong Medical University, Foshan, China

**Keywords:** placenta accreta spectrum disorders, systemic inflammation response index, systemic immune-inflammation index, prenatal diagnosis, risk stratification

## Abstract

**Background:**

Placenta Accreta Spectrum (PAS) disorders are associated with substantial maternal morbidity, and distinguishing mild from severe forms prenatally remains difficult. Inflammatory indices derived from peripheral blood counts have emerged as promising biomarkers, yet their role in grading PAS severity has been insufficiently investigated. This study aimed to assess the discriminative value of the systemic immune-inflammation index (SII), systemic inflammation response index (SIRI), and other inflammatory indices for differentiating severe from mild PAS within an already diagnosed PAS population in late pregnancy.

**Methods:**

This retrospective single-centre cohort study included 1,276 patients with histopathologically confirmed PAS. Third-trimester neutrophil, lymphocyte, monocyte, and platelet counts were used to derive neutrophil-to-lymphocyte ratio (NLR), platelet-to-lymphocyte ratio (PLR), SII, and SIRI. Multivariable logistic regression, receiver operating characteristic curve analysis, and decision curve analysis (DCA) were conducted.

**Results:**

Compared with the mild PAS group (n = 1,060), the severe PAS group (n = 216) showed significantly lower SIRI, SII, NLR, and PLR values (all *P* < 0.001). SIRI demonstrated moderate discriminative performance (area under the curve = 0.712). DCA suggested its potential clinical usefulness across threshold probabilities.

**Conclusions:**

Lower third-trimester SIRI, SII, NLR, and PLR values are associated with severe PAS. SIRI shows moderate diagnostic performance and may serve as a cost-effective adjunct to imaging for prenatal risk stratification. Prospective validation is warranted.

Placenta Accreta Spectrum (PAS) disorders represent one of the most severe complications encountered in contemporary obstetrics [1]. Based on the depth of trophoblastic invasion, these conditions are histologically categorised as placenta accreta, increta, or percreta [2]. PAS can result in life-threatening postpartum haemorrhage, multisystem organ failure, and maternal death [3]. With increasing rates of caesarean deliveries, advancing maternal age, and evolving fertility policies, the incidence of PAS has risen substantially, presenting a major threat to maternal health and placing considerable strain on healthcare systems [4–7].

Accurate subclassification of PAS is critical for optimal clinical decision-making [8,9]. Specifically, distinguishing between abnormally adherent placenta (mild PAS, placenta accreta) and abnormally invasive placenta (severe PAS, encompassing placenta increta and percreta) is essential, as these entities demand markedly different management strategies [10]. A timely and accurate prenatal diagnosis facilitates essential preoperative planning, including determining the optimal timing and setting for delivery, arranging adequate blood product reserves, and assembling a multidisciplinary team experienced in PAS management [11]. Failure to diagnose PAS before delivery is linked to severe maternal complications, largely due to the extensive surgical procedures required to manage the associated haemorrhage [12]. Currently, prenatal diagnosis relies mainly on ultrasound and magnetic resonance imaging (MRI) [13–16]. However, these modalities have inherent limitations in accurately differentiating PAS subtypes, including operator dependence, suboptimal resolution, and subjective interpretation, particularly in cases of posterior placenta or in resource-constrained settings [17,18]. Although several serum biomarkers have been explored, their clinical utility remains constrained by complexity, cost, and a lack of standardisation [19]. These challenges highlight the need for rapid, accessible biomarkers capable of effectively assessing the degree of placental implantation, especially prior to delivery.

Under normal conditions, placental invasion is confined to the inner third of the myometrium through tightly regulated spatial and temporal mechanisms [20]. In PAS, however, the placenta exhibits invasive behaviour, proliferating and invading adjacent tissues in a manner reminiscent of malignant tumours [21,22]. The systemic immune-inflammation index (SII) and systemic inflammation response index (SIRI) are composite biomarkers derived from peripheral blood cell counts, including neutrophils, platelets, lymphocytes, and monocytes, that reflect the systemic inflammatory and immune status [23,24]. Recently, these indices have gained relevance in inflammation-related disorders, certain cancers, and pregnancy-associated disorders [25,26]. Several studies have investigated the association between inflammatory indices and PAS. For example, the neutrophil-to-lymphocyte ratio (NLR), platelet-to-lymphocyte ratio (PLR), SII, and SIRI have been shown to predict PAS in pregnant women with placenta previa [27,28], and NLR may offer additional diagnostic value for caesarean scar pregnancy [29]. However, most existing studies have focused on dichotomous comparisons between patients with and without PAS, with limited attention to subclassification within the PAS spectrum.

A recent study by Karatasli *et al.* evaluated associations between first-trimester screening markers (PAPP-A, β-hCG) and haematological indices in PAS pregnancies complicated by placenta previa, reporting elevated PAPP-A and β-hCG and decreased mean platelet volume in PAS cases, but no significant differences in NLR, PLR, or other inflammatory ratios [30]. That study complements the existing literature by focusing on early pregnancy markers, whereas the present study examines third-trimester inflammatory indices for severity stratification.

To contextualise our findings, we conducted a PubMed search using the terms ‘placenta accreta’ AND (‘inflammatory indices’ OR ‘inflammatory markers’ OR ‘NLR’ OR ‘PLR’ OR ‘SII’ OR ‘SIRI’) to identify prior studies in this field. The present study adds to the growing body of literature by evaluating the performance of composite inflammatory indices in distinguishing PAS severity in a large cohort with histopathological confirmation. We aimed to evaluate the discriminative value of SII, SIRI, and other inflammatory indices in late pregnancy, providing a potentially novel approach for the identification of patients at high risk for severe PAS within an already diagnosed PAS population.

## METHODS

### Study design and population

This retrospective cohort study analysed data from a prospectively maintained database at The Affiliated Foshan Women and Children Hospital, Guangdong Medical University. The Institutional Review Board approved the study protocol (Approval No. FSFY-MEC-2026-003), which adhered to the principles of the Declaration of Helsinki. Owing to the retrospective design and anonymisation of data, the requirement for informed consent was waived.

The study population comprised pregnant women who delivered singleton infants at our institution between January 2021 and December 2025. All participants received a prenatal diagnosis of PAS *via* ultrasound or MRI, which was later confirmed by intraoperative findings and histopathological examination [31,32]. The final diagnosis was established based on intraoperative assessment by experienced obstetricians (loss of the normal cleavage plane, difficulty in placental separation, and associated haemorrhage) and histopathological examination of resected placental-bed tissue specimens.

PAS was classified according to the depth of trophoblastic invasion:

1. placenta accreta, villi attached to the myometrium without intervening decidua;

2. placenta increta, villi invade into the myometrium; and

3. placenta percreta, villi penetrate through the myometrium and serosa or into adjacent organs [12,13].

For analysis, placenta accreta was defined as mild PAS (mPAS), while placenta increta and percreta were combined as severe PAS (sPAS). Of note, among the 216 sPAS patients, 213 (98.6%) had placenta increta and 3 (1.4%) had placenta percreta. The majority of sPAS patients (n = 213, 98.6%) underwent partial myometrial resection with uterine preservation, while three patients (1.4%) with placenta percreta required hysterectomy. All patients had tissue specimens sent for histopathological confirmation.

Inclusion criteria were:

1. confirmed diagnosis of PAS;

2. availability of complete antenatal haematological records; and

3. singleton pregnancy.

Exclusion criteria included acute gestational infection, chronic inflammatory diseases, autoimmune disorders, hematologic malignancies, or incomplete records. A total of 67 patients were excluded for the following reasons: acute infections (n = 34), chronic inflammatory diseases (n = 22), and autoimmune disorders (n = 11).

### Data collection

We extracted demographic characteristics, clinical data, and laboratory results from the hospital’s electronic medical record system. The collected information included medical history, predelivery laboratory findings, and histopathological reports. The following variables were collected: maternal age, gravidity, parity, body mass index (BMI), prior caesarean section, placenta previa status, gestational age at delivery, neonatal birth weight, and neonatal sex. Complete-case analysis was used, as missing data were limited to the 67 excluded patients; no imputation was performed.

### Haematological assessment

All patients underwent standardised laboratory testing before delivery. For haematological assessment, peripheral venous blood samples were obtained at the last routine antenatal visit before delivery. The mean gestational age at blood draw was 38.35 ± 1.76 weeks in the mPAS group and 35.76 ± 2.53 weeks in the sPAS group (*P* < 0.001). Complete blood counts were performed using a SYSMEX XN-2000 Hematology Analyzer (Sysmex Corporation, Japan). Absolute counts of neutrophils, lymphocytes, monocytes, and platelets were recorded. NLR was calculated as the neutrophil count divided by the lymphocyte count, and PLR as the platelet count divided by the lymphocyte count. SII was derived as (platelet count × neutrophil count) / lymphocyte count. SIRI was calculated as (neutrophil count × monocyte count) / lymphocyte count [23,26].

### Statistical analysis

Categorical variables are presented as counts and percentages. Normally distributed continuous variables are expressed as mean ± standard deviation, whereas non-normally distributed variables are presented as median with interquartile range. Group comparisons for categorical variables were performed using χ^2^ tests or Fisher exact tests, as appropriate. For continuous variables, comparisons were made using Student’s *t* test or the Mann-Whitney U test, depending on the distribution.

Logistic regression analysis was used to develop a multivariable prediction model for sPAS risk. The multivariable model adjusted for maternal age, gravidity, parity, gestational age at blood draw, BMI, prior caesarean section, and placenta previa. Each inflammatory index was evaluated in a separate multivariable model (one index per model) to avoid collinearity issues arising from their shared mathematical components. Receiver operating characteristic (ROC) curve analysis was performed to determine cutoff values, sensitivity, and specificity, and the area under the curve (AUC) was calculated to assess diagnostic performance. The Youden index (J = sensitivity + specificity −1) was applied to identify the optimal cutoff point maximising overall diagnostic accuracy. Calibration of the SIRI model was assessed using the Hosmer-Lemeshow goodness-of-fit test and the Brier score, and a calibration plot was generated. Decision curve analysis (DCA) was used to evaluate the clinical utility of the model [33,34]. Multicollinearity among NLR, SII, and SIRI was assessed using variance inflation factor (VIF). A post hoc power calculation indicated that the sample size (216 sPAS cases and 1060 mPAS cases) provided >90% power to detect an AUC of 0.712 (95% CI = 0.669–0.756) as significantly different from a null AUC of 0.65 at α = 0.05 (two-sided), suggesting adequate power for the primary analyses.

To further address potential confounding, we performed three additional analyses:

1. a gestational-age-matched subsample analysis (1:1 matching within ±3 days),

2. an interaction test between severity group and gestational age at blood draw, and

3. a gestational-age-standardised residuals analysis.

Internal validation of the SIRI model was performed using bootstrap resampling with 1000 iterations to obtain optimism-corrected performance estimates. Sensitivity analysis excluding patients with antepartum haemorrhage was also conducted to assess the potential impact of haemodilution on the observed associations. All statistical analyses were conducted using IBM SPSS Statistics for Windows, version 21 (IBM Corp., Armonk, NY, USA), Stata Statistical Software, Release 17 (StataCorp LLC, College Station, TX, USA) and *R*, version 4.3.2. (R Core Team, R Foundation for Statistical Computing, Vienna, Austria, 2023). All tests were two-sided, and a *P*-value <0.05 was considered statistically significant.

## RESULTS

### Baseline characteristics

A total of 1,276 patients met the inclusion criteria, consisting of an mPAS group (n = 1,060) and an sPAS group (n = 216). Baseline characteristics are summarised in **Table 1**. Patients in the sPAS group were significantly older (34.23 ± 4.94 *vs.* 30.71 ± 4.51 years, *P* < 0.001) and had higher gravidity and parity compared with the mPAS group (both *P* < 0.001). Patients in the sPAS group also had significantly higher rates of prior caesarean section (67.1% *vs.* 29.1%, *P* < 0.001) and placenta previa (54.6% *vs.* 8.3%, *P* < 0.001). BMI did not differ significantly between the two groups (25.27 ± 2.14 *vs.* 24.96 ± 4.53 kg/m^2^, *P* = 0.757). Regarding neonatal outcomes, the sPAS group had a notably lower mean gestational age at delivery (36.27 ± 2.53 *vs.* 38.83 ± 1.75 weeks, *P* < 0.001) and lower birth weight (2728.36 ± 593.79 *vs.* 3091.79 ± 479.86 g, *P* < 0.001). No significant difference was observed in the proportion of male newborns between the groups (*P* = 0.654).

**Table 1:** Baseline characteristics and inflammatory indexes in mPAS and sPAS patients

	mPAS (n = 1,060)	sPAS (n = 216)	t/χ^2^	*P*-value	OR (95% CI)*	*P*-value*
Maternal age (year)	30.71 ± 4.51	34.23 ± 4.94	9.69	<0.001		
BMI	25.27 ± 2.14	24.96 ± 4.53	0.637	0.757		
Gravidity, n (%)						
*1*	381 (35.9)	17 (7.9)	66.826	<0.001		
*2*	392 (37.0)	108 (50.0)				
*≥3*	287 (27.1)	91 (42.1)				
Parity, n (%)						
*0*	509 (48.0)	30 (13.9)	93.654	<0.001		
*1*	449 (42.4)	136 (63.0)				
*≥2*	102 (9.6)	50 (23.1)				
Prior cesarean section, n (%)	308 (29.1)	145 (67.1)	113.592	<0.001		
Placenta previa, n (%)	88 (8.3)	118 (54.6)	284.473	<0.001		
Gestational age at delivery (week)	38.83 ± 1.75	36.27 ± 2.53	13.892	<0.001		
Gestational age at blood draw (week)	38.35 ± 1.76	35.76 ± 2.53	14.018	<0.001		
Birth weight (g)	3,091.79 ± 479.86	2,728.36 ± 593.79	8.451	<0.001		
Male newborn, n (%)	566 (53.4)	119 (55.1)	0.208	0.654		
Neutrophil (10^9^/L)	8.33 ± 2.36	7.00 ± 2.37	7.560	<0.001	0.710 (0.645–0.782)	<0.001
Monocyte (10^9^/L)	0.76 ± 0.21	0.66 ± 0.25	5.920	<0.001	0.117 (0.047–0.291)	<0.001
Lymphocyte (10^9^/L)	1.64 ± 0.49	1.63 ± 0.50	0.264	0.792	1.186 (0.827–1.702)	0.354
Platelet (10^9^/L)	239.25 ± 58.35	223.95 ± 57.07	3.524	<0.001	0.997 (0.994–1.000)	0.091
NLR	5.52 ± 2.58	4.65 ± 2.29	4.609	<0.001	0.745 (0.676–0.822)	<0.001
PLR	156.31 ± 55.21	146.19 ± 48.04	2.749	0.006	0.995 (0.991–0.999)	0.007
SII	1,306.32 ± 656.45	1,022.69 ± 503.83	7.132	<0.001	0.999 (0.998–0.999)	<0.001
SIRI	4.12 ± 2.13	2.96 ± 1.70	8.708	<0.001	0.577 (0.496–0.671)	<0.001

BMI – body mass index, CI – confidence interval, Mpas – mild placenta accreta spectrum disorders, NLR – neutrophil-to-lymphocyte ratio, PLR – platelet-to-lymphocyte ratio, OR – odds ratio, SII – systemic immune-inflammation index, SIRI – systemic inflammation response index, sPAS – severe placenta accreta spectrum disorders

*Adjusted for maternal age, gravidity, parity, gestational age at blood draw, BMI, prior cesarean section, and placenta previa.

### Inflammatory indices

The sPAS group exhibited significantly lower absolute counts of neutrophils, monocytes, and platelets compared with the mPAS group (all *P* < 0.001), whereas lymphocyte counts were similar between groups (*P* = 0.792). Consequently, NLR, PLR, SII, and SIRI were all significantly lower in the sPAS group than in the mPAS group (all *P* < 0.001). After adjusting for maternal age, gravidity, parity, gestational age at blood draw, BMI, prior caesarean section, and placenta previa in multivariable logistic regression analysis, neutrophil count, monocyte count, NLR, PLR, SII, and SIRI remained significantly associated with a reduced likelihood of sPAS (**Table 1**). We note that these inflammatory indices share mathematical components and are not independent; each index was therefore evaluated in a separate multivariable model to avoid collinearity issues. When expressed per standard-deviation (SD) increase (SIRI SD = 2.10), each SD increase in SIRI was associated with a 42% reduced likelihood of sPAS (OR = 0.58; 95% CI = 0.50–0.67).

### Diagnostic performance

We evaluated the predictive performance of inflammatory parameters for sPAS using ROC curve analysis. The Youden index was applied to identify cutoff values that optimise the balance between sensitivity and specificity for diagnosing sPAS (**Figure 1**, **Table 2**). The AUC values, ranked from highest to lowest, were as follows: SIRI (0.712), neutrophil count (0.675), SII (0.655), monocyte count (0.644), NLR (0.639), platelet count (0.574), PLR (0.550), and lymphocyte count (0.516) (**Table 2**). Pairwise comparison of AUCs using the DeLong test showed that SIRI was significantly superior to platelet count (*P* = 0.023), PLR (*P* = 0.012) and lymphocyte count (*P* < 0.001), but not significantly different from neutrophil count (*P* = 0.187), monocyte count (*P* = 0.376), NLR (*P* = 0.512) or SII (*P* = 0.694). The optimal cutoff value for SIRI was 2.272, yielding a sensitivity of 98.2% and a specificity of 39.8%. At the study prevalence of severe PAS (16.9%), the positive predictive value (PPV) was 24.9% and the negative predictive value (NPV) was 99.1%, indicating that SIRI is a strong rule-out tool but a weak rule-in test.

**Figure 1:**
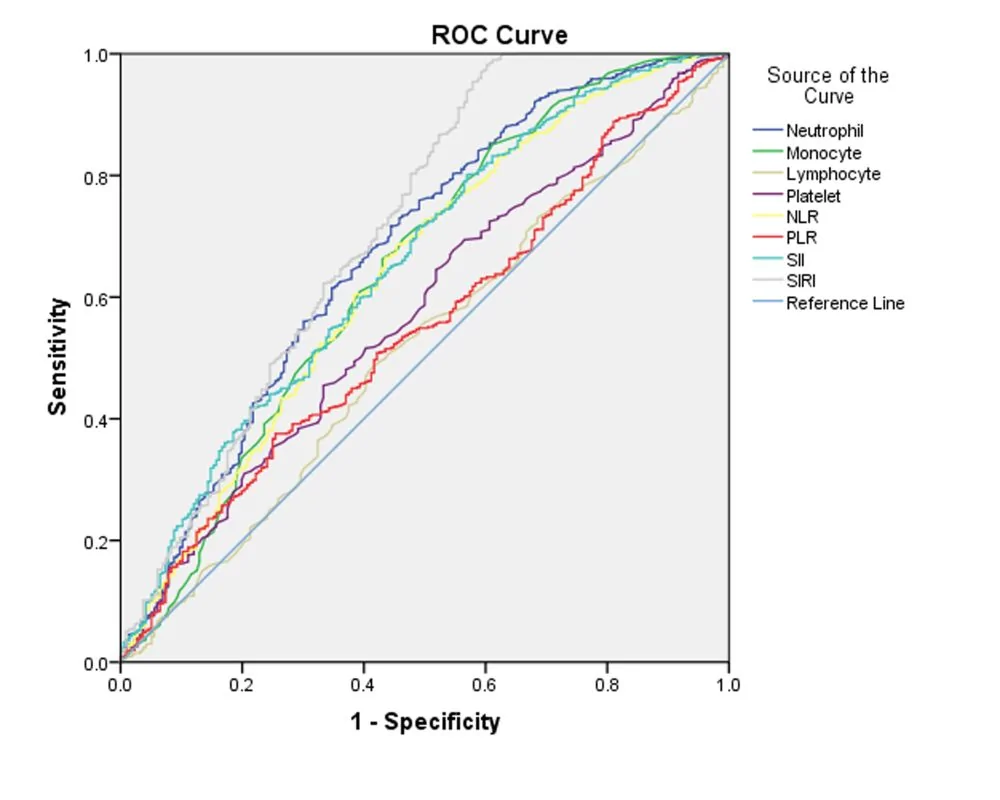
ROC curves of inflammatory indices for predicting sPAS. NLR – neutrophil-to-lymphocyte ratio, PLR – platelet-to-lymphocyte ratio, ROC – receiver operating characteristic, SII – systemic immune-inflammation index, SIRI – systemic inflammation response index, sPAS – severe placenta accreta spectrum.

**Table 2:** ROC curve analysis of inflammatory indices in the prediction of sPAS

	Cut-off value	Sensitivity	Specificity	AUC	95% CI	*P*-value
Neutrophil (10^9^/L)	6.845	0.717	0.556	0.675	0.633–0.717	<0.001
Monocyte (10^9^/L)	0.565	0.851	0.389	0.644	0.600–0.688	<0.001
Lymphocyte (10^9^/L)	1.615	0.487	0.583	0.516	0.473–0.558	0.464
Platelet (10^9^/L)	210.500	0.676	0.454	0.574	0.533–0.616	<0.001
NLR	4.235	0.686	0.546	0.639	0.597–0.682	<0.001
PLR	163.022	0.375	0.745	0.550	0.509–0.591	0.020
SII	846.021	0.801	0.431	0.655	0.614–0.697	<0.001
SIRI	2.272	0.982	0.398	0.712	0.669–0.756	<0.001

AUC – area under the ROC curve, CI – confidence interval, NLR – neutrophil-to-lymphocyte ratio, PLR – platelet-to-lymphocyte ratio, ROC – receiver operating characteristic, SII – systemic immune-inflammation index, SIRI – systemic inflammation response index, sPAS – severe placenta accreta spectrum disorders

Calibration of the SIRI model was assessed using the Hosmer-Lemeshow test, which yielded a *P* value of 0.382, indicating good calibration. The Brier score was 0.0539, and the calibration plot (**Figure 2**) demonstrated acceptable agreement between predicted and observed probabilities. Multicollinearity assessment showed VIF values of 4.234 for NLR, 3.663 for SII, and 2.403 for SIRI, indicating no significant multicollinearity among the inflammatory indices.

**Figure 2:**
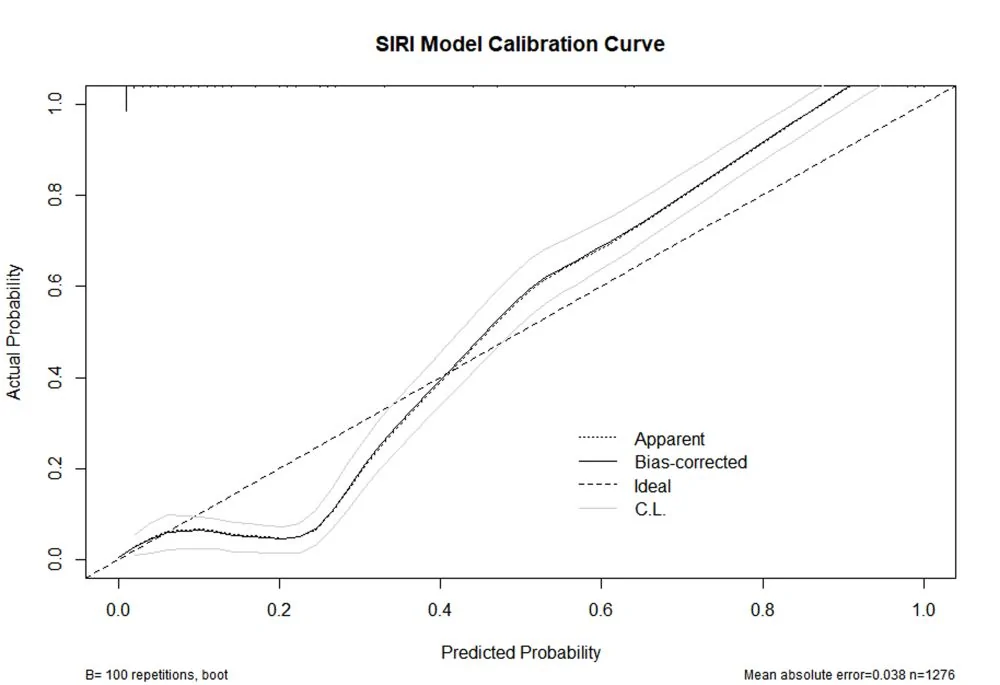
Calibration plot of the SIRI model. CL – confidence limit, SIRI – systemic inflammation response index.

Decision curve analysis further demonstrated the net benefit of the predictive model based on inflammatory markers, with the SIRI model showing modest superior performance (**Figure 3**). At a threshold probability of 0.10, the SIRI model yielded a net benefit of 0.0772, comparable to that of the ‘treat-all’ strategy (0.0770). At higher thresholds (0.20 and 0.30), the SIRI model outperformed the ‘treat-all’ strategy, maintaining a positive net benefit while the latter declined to negative values (**Table 3**).

**Figure 3:**
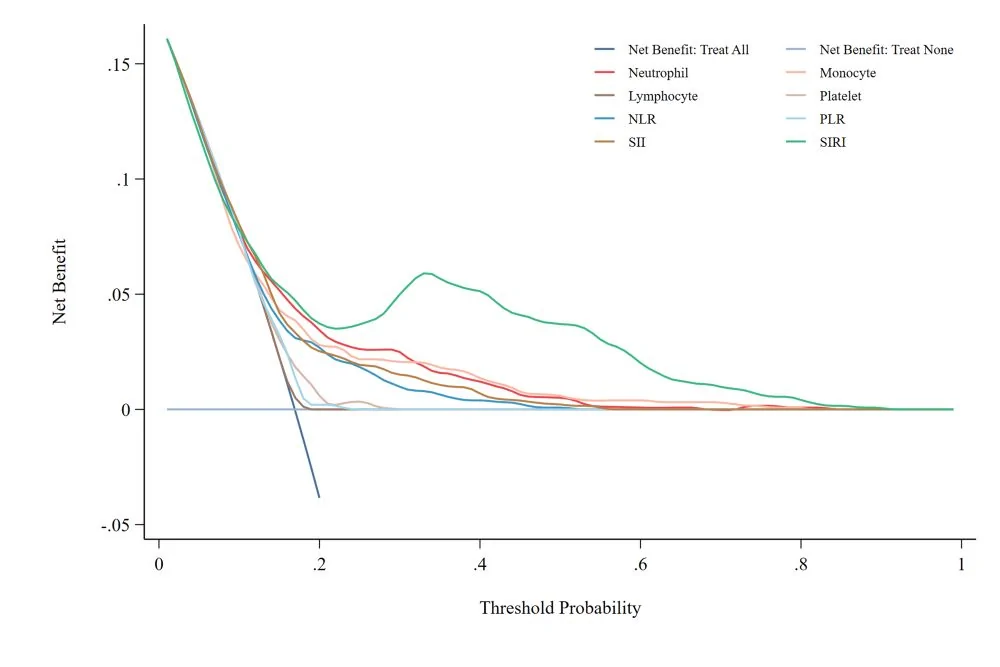
Decision curve analysis for predicting the probability of sPAS. NLR – neutrophil-to-lymphocyte ratio, PLR – platelet-to-lymphocyte ratio, SII – systemic immune-inflammation index, SIRI – systemic inflammation response index, sPAS – severe placenta accreta spectrum disorders.

**Table 3:** Clinical net benefit analysis of the SIRI prediction model

Threshold	SIRI_NB	Treat_All_NB	Net_Benefit_Increase
0.1	0.0772	0.0770	0.0003
0.2	0.0343	−0.0384	0.0727
0.3	0.0525	−0.1867	0.2393

SIRI – systemic inflammation response index

### Sensitivity and validation analyses

As previously described, we performed three additional analyses to address potential confounding by gestational age at blood draw (matched subsample, interaction test, and standardised residuals). In a gestational-age-matched subsample (1:1 matching within ±3 days, n = 432), the association between SIRI and severe PAS remained significant (OR = 0.67; 95% CI = 0.59–0.78) (Table S1 in the **Online Supplementary Document**). A formal test of interaction between severity group and gestational age at blood draw was not significant (*P* = 0.155), indicating no evidence of effect modification. Gestational-age-standardised residuals for SIRI remained significantly different between groups (*P* < 0.001).

To address the possibility that haemodilution from antepartum haemorrhage might explain the observed associations, we performed a sensitivity analysis excluding 326 patients with documented antepartum haemorrhage. The inverse association between SIRI and severe PAS remained significant (OR = 0.63; 95% CI = 0.54–0.74). An interaction term between severity group and haemorrhage status was not significant (*P* = 0.376), suggesting that the observed associations are not solely attributable to haemorrhage-related haemodilution (Table S1 in the **Online Supplementary Document**).

To address the optimism bias inherent in deriving a cutoff and testing it on the same data set, we performed bootstrap internal validation with 1000 resamples. The optimism-corrected AUC for SIRI was 0.713 (95% CI = 0.662–0.763), with optimism-corrected sensitivity and specificity of 0.941 and 0.372, respectively (Table S1 in the **Online Supplementary Document**).

## DISCUSSION

In this retrospective cohort study of 1276 patients with histopathologically confirmed PAS, we observed that systemic inflammatory indices, specifically SIRI, SII, NLR, and PLR, measured during the third trimester were significantly lower in patients with severe PAS (placenta increta and percreta) compared with those with mild PAS (placenta accreta). Among these markers, SIRI demonstrated moderate discriminatory ability for identifying severe PAS, with an AUC of 0.712. The Hosmer-Lemeshow test (*P* = 0.382) and Brier score (0.0539) indicated acceptable calibration of the SIRI model. DCA suggested its potential clinical utility across threshold probabilities. This study adds to the growing body of literature by evaluating the performance of composite inflammatory indices in distinguishing PAS severity in a large cohort with histopathological confirmation, and by providing calibration metrics for the SIRI prediction model.

We acknowledge that the inverse association observed in this study is counterintuitive, as elevated inflammatory indices are conventionally interpreted as indicators of greater disease severity. One plausible hypothesis is that severe PAS is characterised by localised immune tolerance at the maternal-foetal interface, which may be associated with attenuated systemic inflammatory responses [35]. However, we emphasise that this interpretation remains speculative and should not be used to infer causality. Alternative explanations should also be considered, including:

1. potential corticosteroid exposure (though we lacked data on antenatal steroid administration),

2. chronic blood loss and haemodilution in patients with antepartum haemorrhage, which may occur more frequently in severe PAS, and

3. differences in gestational age at blood sampling (the sPAS group had earlier blood draws, which we adjusted for in the multivariable model) [36–39].

Our findings are generally consistent with prior studies reporting associations between NLR, PLR, and SII and the presence of PAS in high-risk populations, such as those with placenta previa or caesarean scar pregnancy [27–29]. However, most previous work has focused on dichotomous comparisons between patients with and without PAS, rather than on subclassification within the PAS spectrum. A recent study by Karatasli *et al.* evaluated first-trimester screening markers and haematological indices in PAS pregnancies complicated by placenta previa, reporting elevated PAPP-A and β-hCG and decreased mean platelet volume in PAS cases, but no significant differences in NLR, PLR, or other inflammatory ratios [30]. That study differs from ours in timing (first-trimester *vs.* third-trimester), population (placenta previa-restricted *vs.* all PAS), and outcome (PAS presence *vs.* PAS severity). Taken together, these findings suggest that early and late inflammatory markers may capture different aspects of PAS pathophysiology, and future studies integrating both time points may yield more comprehensive risk stratification tools.

The present study extends the literature by revealing that within PAS-affected pregnancies, more severe invasive phenotypes are paradoxically associated with lower values of these indices. This counterintuitive pattern underscores the importance of distinguishing between ‘adherent’ and ‘invasive’ forms of PAS, as their underlying biological mechanisms may differ substantially.

From a clinical perspective, these easily obtainable, cost-effective markers offer a potential adjunct to imaging for prenatal risk stratification. Current imaging modalities are operator-dependent and subject to interpretative variability, particularly in resource-limited settings or for posterior placental locations [17,18]. Given the high sensitivity (98.2%) but low specificity (39.8%) of SIRI at the optimal cutoff value of 2.272, this marker may be more useful as a screening/rule-out tool rather than a confirmatory test. Patients with low SIRI values may warrant closer surveillance or referral to specialised centres, while abnormal SIRI values alone should not be used to confirm severe PAS without imaging confirmation. Incorporating serum biomarkers could enhance diagnostic accuracy, facilitating more informed preoperative planning and timely referral to specialised centres equipped to manage severe PAS.

We acknowledge that the nearly 2.6-week difference in gestational age at blood draw between groups represents a potential source of confounding. Although we adjusted for this in the multivariable model and performed additional analyses, including a matched subsample, interaction testing, and standardised residuals, residual confounding cannot be entirely excluded. The consistency of our findings across these sensitivity analyses, however, supports the robustness of the observed inverse association.

Several limitations warrant acknowledgment. The single-centre, retrospective design, conducted in a specialised referral setting with a high rate of uterus-preserving management even among increta/percreta cases (98.6%), may introduce selection bias and limit generalisability to less specialised centres where hysterectomy is more commonly performed. Our findings should therefore be interpreted in this context and validated in diverse settings. We defined severity based on histopathological criteria, which is the gold standard for PAS classification, though we acknowledge that clinical outcomes such as blood loss and hysterectomy represent another important dimension of severity. Although we adjusted for multiple confounders (maternal age, gravidity, parity, gestational age at blood draw, BMI, prior cesarean section, and placenta previa), unmeasured confounders, including variations in clinical management and medication use, cannot be entirely excluded. The small number of percreta cases (n = 3) precluded meaningful subgroup analysis across the invasion spectrum; consequently, our findings primarily reflect differences between placenta accreta and placenta increta, rather than a comprehensive comparison across the full invasion spectrum. Future studies with larger samples are needed to determine whether inflammatory profiles differ between increta and percreta. Additionally, the lack of an external validation cohort precludes confirmation of model robustness; while bootstrap internal validation suggested acceptable optimism-corrected performance, external validation in independent cohorts is needed. The moderate discriminative performance (AUC = 0.712) suggests that SIRI alone is insufficient for definitive diagnosis and should be used in conjunction with clinical and imaging assessments.

Future research should focus on prospective, multicentre validation of the SIRI-based model in diverse populations, as well as exploration of the underlying immunological mechanisms linking systemic inflammatory profiles to the depth of placental invasion. Integrating inflammatory indices with imaging findings and clinical risk factors, potentially using machine learning approaches, may yield more robust predictive algorithms for individualised risk assessment. Longitudinal studies examining the temporal changes in these markers throughout pregnancy could also provide insights into the optimal timing of biomarker assessment. Comparative studies examining both first-trimester and third-trimester markers in the same cohort, as suggested by the work of Karatasli *et al.*, would be particularly valuable [30].

## CONCLUSIONS

Our results suggest that lower third-trimester SIRI, SII, NLR, and PLR values are associated with severe PAS phenotypes, with SIRI demonstrating moderate diagnostic performance. Although the observed associations were statistically significant, the diagnostic accuracy is moderate, and the proposed immune tolerance mechanism remains speculative. These easily obtainable, cost-effective markers may serve as valuable adjuncts to imaging for refining prenatal risk stratification in PAS, particularly as a screening tool given the high sensitivity of SIRI. The SIRI cutoff of 2.272, with its high sensitivity (98.2%) but low specificity (39.8%), may serve as a ‘rule-out’ screening tool: patients with SIRI values above this threshold are unlikely to have severe PAS and may not require escalation of care, whereas those with values below the threshold warrant closer surveillance and multidisciplinary planning. However, we emphasise that these recommendations require prospective validation before clinical implementation and should not yet be used to guide clinical decisions independently of imaging and clinical assessment. If validated in future prospective, multicentre studies, their incorporation into clinical practice could facilitate timely referral to specialised centres and ultimately contribute to improved maternal outcomes in this high-risk obstetric population.

## Additional material


Online Supplementary Document

## Data Availability

**Data availability:** The data sets used and/or analysed during the current study are available from the corresponding author on reasonable request.
