# Supplementary material for: Systemic inflammatory indices for predicting severe placenta accreta spectrum: a retrospective cohort study
Source: J Glob Health. 2026 Oct 19;16:04353. doi: 10.7189/jogh.16.04353 (PMC13679349; doi:10.7189/jogh.16.04353)
Supplement: Supplementary file 1 [file jogh-16-04353-s001.pdf]

Supplement to: Lu X, Lei X, Guo C, Tang Q, Zhang X, Mai W, Mo M, Yuan M, Wang W, Fan D. Systemic inflammatory indices for predicting severe placenta accreta spectrum: a retrospective cohort study. J Glob Health. 2026;16:04353.

Table S1. Sensitivity and Validation Analyses for the SIRI Model.

	AUC	95%CI	p-value	OR (95%CI)*	p-value*
Matched subsample	0.724	0.676-0.772	0.001	0.677 (0.588-0.779)	< 0.001
Excluding antepartum hemorrhage	0.704	0.653-0.755	0.001	0.633 (0.539-0.743)	< 0.001
Internal validation	0.713	0.662-0.763	0.001	0.639 (0.545-0.749)	< 0.001

AUC: area under the ROC curve; CI: confidence interval; ROC: receiver operating characteristic; SIRI: systemic inflammation response index.
*Adjusted for maternal age, gravidity, parity, BMI, prior cesarean section, and placenta previa. OR for internal validation represents optimism-corrected estimate from bootstrap.
